# What do the *JAMA *editors say when they discuss manuscripts that they are considering for publication? Developing a schema for classifying the content of editorial discussion

**DOI:** 10.1186/1471-2288-7-44

**Published:** 2007-09-25

**Authors:** Kay Dickersin, Elizabeth Ssemanda, Catherine Mansell, Drummond Rennie

**Affiliations:** 1 Department of Epidemiology, Johns Hopkins Bloomberg School of Public Health, 615 North Wolfe Street, Baltimore, Maryland 21205, USA; 2 Providence, Rhode Island, USA; 3 Deputy Editor *JAMA*, The Institute for Health Policy Studies, University of California San Francisco, San Francisco, California, USA

## Abstract

**Background:**

In an effort to identify previously unrecognized aspects of editorial decision-making, we explored the words and phrases that one group of editors used during their meetings.

**Methods:**

We performed an observational study of discussions at manuscript meetings at *JAMA*, a major US general medical journal. One of us (KD) attended 12 editorial meetings in 2003 as a visitor and took notes recording phrases from discussion surrounding 102 manuscripts. In addition, editors attending the meetings completed a form for each manuscript considered, listing the reasons they were inclined to proceed to the next step in publication and reasons they were not (DR attended 4/12 meetings). We entered the spoken and written phrases into NVivo 2.0. We then developed a schema for classifying the editors' phrases, using an iterative approach.

**Results:**

Our classification schema has three main themes: science, journalism, and writing. We considered 2,463 phrases, of which 87 related mainly to the manuscript topic and were not classified (total 2,376 classified). Phrases related to science predominated (1,274 or 54%). The editors, most of whom were physicians, also placed major weight on goals important to JAMA's mission (journalism goals) such as importance to medicine, strategic emphasis for the journal, interest to the readership, and results (729 or 31% of phrases). About 16% (n = 373) of the phrases used related to writing issues, such as clarity and responses to the referees' comments.

**Conclusion:**

Classification of editorial discourse provides insight into editorial decision making and concepts that need exploration in future studies.

## Background

No public knowledge is gained from scientific and biomedical research unless the study methods and results are properly written up and disseminated, typically by publication in a widely available journal. The decision to publish study findings involves many individuals and groups, including the investigators, the designated authors, the peer reviewers, the journal editors, and editor-in-chief. Some parts of the decision-making process are more easily studied than others, in part because traditionally, publishing decisions have been confidential. From substantial existing research, we know that investigators, not journal editors, are the ones mainly responsible for the decision to publish, and that this decision is related to the direction and strength of study findings (publication bias) [1-6].

There are many fewer studies of the editorial decision-making process [7-10]. Editors cannot discuss or make decisions about manuscripts they never receive. A 2002 study of editorial decision-making at the US general medical journal *JAMA *found that editors favor high quality science, and any bias against negative results is either small or does not exist [7]. Studies of editorial decision-making have mainly addressed issues of bias, but not other factors related to journal mission, responsibility, and financial health. For example, one might ask whether editors are interested in publishing "newsworthy" results: those that are completely unexpected, that confirm a long held belief, or that settle a controversy once and for all. One might also be interested in whether a single set of rules could be devised to describe acceptance decisions. Editors may one week reject a report because it is similar to another one recently published, but may the next week accept a paper because it is similar to a recent contribution in their journal.

*JAMA*, a major, weekly, general medical journal in the United States, has a broad mission and low acceptance rate. We decided to attend journal editorial decision-making meetings to learn more about what was discussed, to allow construction of quantitative survey instruments that would capture a richer picture of factors influencing publication decisions. The discussions we listened to relate particularly to understanding the mechanisms used when editors must choose only 5% to 10% from a large pool of submitted manuscripts.

In an effort to identify previously unrecognized aspects of editorial decision-making, we explored the words and phrases that one group of editors used during their meetings. We did not use formal qualitative methods to analyze the information collected, rather our goal was to help to generate hypotheses for those doing research on editorial decision making and publication practices.

## Methods

In 2003, 5064 manuscripts (not including letters) were submitted to *JAMA*. These were distributed to the editors, almost all physicians, who reject at least half outright without obtaining comments from peer reviewers. When manuscripts are returned from peer reviewers, typically two or three, editors can reject directly or bring the manuscript to an editorial meeting. Some editors bring most of their papers to the table for a decision and others bring only papers they feel have a very good chance of acceptance. Because manuscripts are assigned to a single editor for review, only one editor has typically read a manuscript before the meeting. In 2003, approximately 940 manuscripts were brought to the meeting for discussion. All discussions at the meetings are kept in strict confidence, by JAMA's standing rules.

One of us (KD) attended 12 twice weekly editorial meetings, as a visitor, at the *JAMA *offices in Chicago, Illinois, USA in January and February 2003 (some meetings were missed by KD because of scheduling conflicts), and took notes on the discussion surrounding 102 manuscripts (two related manuscripts were discussed as one, and so we counted them as a single manuscript). Her notes were not verbatim transcripts of the meeting's discussions. The meeting attendees varied somewhat from meeting to meeting, but typically comprised about eight in-house editors, including editors with content, managing, and statistical responsibilities, and the Editor-in-Chief. Other editors (including DR who attended 2 of the 12 meetings in person, and 2 by phone) attended by teleconference if a manuscript for which they were responsible was being discussed. Editors volunteered one-by-one, but in no particular order, to discuss the manuscripts for which they were responsible. Anecdotal reports from editors attending the manuscript meetings is that the meetings attended as part of this study were representative of other meetings.

The discussion of each manuscript began with a description of the paper topic and study characteristics, with details added as necessary. The presentation progressed to comments made by the peer reviewers. The vast majority of presented manuscripts had completed at least one round of peer review.

The note-taker (KD) recorded words and phrases spoken by the editors in the context of each manuscript discussed, the time each discussion took, and the comments and publication recommendation of each of the peer reviewers. In addition, at the end of discussion about each manuscript, editors attending the meeting completed a form on which they listed the reasons they were "inclined to proceed to the next step towards review and/or acceptance" and reasons they were not. The editors were asked not to record their names on these forms. Forms were not collected at meetings KD did not attend.

ES and CM extracted the phrases from KD's notes and from the completed forms and entered them into NVivo 2.0 qualitative analysis software (Qualitative Solutions and Research Pty. Ltd, Australia). Each manuscript was considered a separate document with an array of attributes such as date of discussion, number of positive and negative reviews by outside peer reviewers, time taken for discussion, its categorization by the editors as describing research or not, and final disposition of the manuscript.

We next used an iterative process to develop a classification schema for the 2,463 spoken and written phrases. We considered several possible themes, including the *JAMA *objectives [see Table 1], general journalistic goals, and *ad hoc *schema suggested by the phrases recorded.

**Table 1:** JAMA's Key and Critical Objectives

**Key Objective**	
	To promote the science and art of medicine and the betterment of the public health

**Critical Objectives**	

1.	To maintain the highest standards of editorial integrity independent of any special interests
2.	To publish original, important, well-documented, peer-reviewed articles on a diverse range of medical topics
3.	To provide physicians with continuing education in basic and clinical science to support informed clinical decisions
4.	To enable physicians to remain informed in multiple areas of medicine, including developments in fields other than their own
5.	To improve health and health care internationally by elevating the quality of medical care, disease prevention, and research
6.	To foster responsible and balanced debate on issues that affect medicine and health care
7.	To anticipate important issues and trends in medicine and health care
8.	To inform readers about nonclinical aspects of medicine and public health, including the political, philosophic, ethical, legal, environmental, economic, historical, and cultural
9.	To recognize that, in addition to these specific objectives, THE JOURNAL has a social responsibility to improve the total human condition and to promote the integrity of science
10.	To achieve the highest level of ethical medical journalism and to produce a publication that is timely, credible, and enjoyable to read

For the first draft of the schema, CM and KD reviewed the phrases and documents from the editors' discussions and assigned them to 20 categories related to medical editorial decision-making and publication bias (as defined above). This schema was reviewed by two independent epidemiologists. Each performed an independent review followed by a group discussion with KD. We modified the schema further, categorizing phrases by whether they were related to science (eg, likelihood of bias relating to the study design), editorial beliefs or values (eg, likely interpretation by the public), or manuscript features (eg, short, well written). Using Nvivo, CM re-sorted most of the 20 categories into the new schema, retiring some categories and merging others. In a one hour meeting, CM and KD presented the revised schema to two independent social scientists, who suggested additional refinements.

Finally, we revised the concept of editorial beliefs and values to encompass what we called general journalistic goals, and developed a separate construct within the schema. We considered journalism in medicine to encompass a broad mission that includes educational, public health, and strategic goals such as timeliness, serving the readers' interests, presenting important medical issues, and addressing controversies. In the present instance, "journalism goals" were those that spoke to the mission of JAMA and other medical journals – meaning factors and values important to medical (or clinical) journals that publish new research (such as importance to medicine, strategic emphasis for the journal, and interest to the readership).

Thus, we classified each phrase as belonging to one of three mutually exclusive categories: science, journalism, and writing. Each phrase was further classified using a subcode within each category and each phrase was assigned a single code. All categories include phrases that are both favorable and unfavorable, although we describe the category using mainly positive terms [see Table 2]. For instance, phrases used to note exemplary ethical processes, as well as phrases used to note suspected conflict of interest, were classified as part of the "Ethics/Conflict of Interest" category.

**Table 2:** Classification Schema for 2376 Written and Spoken Phrases

**A. Science **(1274 phrases)		
**1.**	**Research Design and Methods **(481 phrases)	Aspects of the quality of the description of the design and methods as well as the methods themselves, from the hypothesis, to assumptions, to bias, to outcomes defined. Also includes some factual statements, such as the type of study design used.
**2.**	**Presentation and Interpretation of Results **(250 phrases)	Concern about message, tone, context, scholarship, rationale for conclusions drawn, whether conclusions match study strength, adequacy of references, preliminary or early results, need for additional discussion.
**3.**	**Analysis **(157 phrases)	Analysis is appropriate, inappropriate, incorrect, incomplete, not clear; rates and measures used. Adjustment for confounders and effect modifiers, issues related to subgroup analysis, intention to treat analysis.
**4.**	**Population Studied **(97 phrases)	Concerns about the population or database and why it was chosen, more information about population needed, potential for selection bias, response rate.
**5.**	**Power, Sample Size **(66 phrases)	Any comment about power, size of study sample or the dataset.
**6.**	**Measures Used **(70 phrases)	Methods specifically concerning measures used and whether they are reliable, valid, are expressed correctly, are sufficient.
**7.**	**More Information/Data Needed **(60 phrases)	Comments that data or details about data are needed, including full CONSORT data.
**8.**	**Generalizable information/results **(37 phrases)	Potential application of information beyond study population, representativeness of broader patient population.
**9.**	**Quality of the Data **(31 phrases)	Concerns about data quality, validity of data, completeness.
**10.**	**Conflict of Interest/Ethics **(25 phrases)	Concerns about financial and other conflicts of interest, as well as concerns about study ethics.

**B. Journalism **(729 phrases)		

**1.**	**Important for Medicine **(131 phrases)	Any statement that the study, results, topic, disease, message are important, of interest, or common.
**2.**	**Strategic Advantage to Publish **(126 phrases)	Reasons why it may be good to publish this paper or not (eg, published elsewhere, previous or current JAMA similar or related publication, need more like this). Specific mention of *JAMA *(except readership) or related to *JAMA *mission. Specialized paper (as opposed to specialized audience), should be sent to Archives. Possible editorial.
**3.**	**Interesting results or topic **(95 phrases)	Any phrase with "interesting" or similar word in it (eg, interesting topic, analysis, hypothesis), except "readers would find interesting". Includes comments relating to a low likelihood of publication leading to behavior change, or the fact that the paper doesn't add anything. Also includes fascinating, boring, good, excellent, great, relevant.
**4.**	**Results (topic) **(76 phrases)	Any statement describing the study outcomes and results.
**5.**	**Author repute **(71 phrases)	References to the author prominence or characteristics of the author (including where from, good group, published previously in *JAMA*), author role in paper.
**6.**	**Positive or negative results **(48 phrases)	Concerns positive, negative or null results or findings of study, statistical significance, beneficial outcomes, positive, negative, modest association, clinically significant, effective intervention, size of association, and effect size.
**7.**	**Novel, new **(47 phrases)	Manuscript contains novel information, findings, data, is a novel study, uses novel methods. New information, unique data, innovation in some aspect of study, new methods, cool new technology, unique work, few similar studies.
**8.**	**Readership **(29 phrases)	Potential readership interest or lack of it, our readers would find it interesting, potential audience beyond medical community, public health interest, policy and government issues, correlation with current events.
**9.**	**Knowledge Gap **(27 phrases)	More information needed in this area.
**10.**	**Timeliness **(24 phrases)	"Hot" or timely topic, topical, big stuff, causes excitement, enormous public health, political interest, pressing issue, increasingly discussed, emerging treatment or drug, problem, increasing disease incidence.
**11.**	**Refutes-Confirms Standard Practice/Thinking **(23 phrases)	Results that refute or confirm standard thinking or practice. Provides new information about clinical problem.
**12.**	**Policy, message and public response **(20 phrases)	Public and private sector response to the topic or findings, possible consequences of publication, including policy implications. Manuscript from government or policy makers.
**13.**	**Special Study Characteristics **(12 phrases)	A characteristic of the study that sets it apart from other studies.

**C. Writing **(373 phrases)		

**1.**	**Peer Review **(282 phrases)	Any comment about revisions needed, peer reviewer comments received, editor's own tendency to accept or reject. When a specific change is requested (eg, "still fuzzy about search strategy"), code with science or journalism, as appropriate.
**2.**	**Writing **(91 phrases)	Relates to presentation only, not clarity of description of study methods.

All categories include positive and negative phrases. For the most part, phrases used here are descriptive and do not reflect exact oral or written statements.

We exported 2,463 coded phrases into Excel 2003 (Microsoft Corp) for counts. We excluded 87 phrases (76 of which were spoken) that were coded as describing the title or topic of the manuscript, leaving 2376 coded phrases for analysis.

Because our goal was to develop new ways to assess manuscript decision-making and publication bias, and because it could in no way influence the fate of manuscripts being discussed, our project did not involve written informed consent. Editors received written material describing the project, and the project was thoroughly discussed, at the initial manuscript meeting before note-taking and form completion began. We consulted officials at the Johns Hopkins Bloomberg School of Public Health Committee on Human Research who requested that we not include identifying information about the editors, peer reviewers, or authors.

## Results

Our results include 2,376 phases, 1,773 spoken and 603 written, which we present combined unless otherwise noted. Spoken phrases include 459 referring specifically to comments made by the peer reviewers and 1,314 that reflect the editors' own comments.

The most frequent phrases used concerned scientific issues (1,274 phrases), including research design and methods, presentation and interpretation of results, analysis methods, population studied, power and sample size, measurement variables and measures used, the need for additional data, generalizability, quality of the data, and conflict of interest/ethics [see Table 2]. Phrases associated with journalistic concerns (n= 729) included those classified as referring to some aspect of the manuscript as important to medicine, associated with a strategic emphasis for the journal, an interesting topic or results, a statement describing the study findings, relating to the author, a study's positive or negative results, novel/new content, readership concerns, filling a knowledge gap, timeliness, refuting or confirming standard practice or thinking, a topic relating to policy issues/a public message/public response, and special study characteristics. Finally, phrases we classified as concerned with writing (n = 373) were coded as either related directly to writing (eg, "not clear", "dense", "needs a rewrite"), or indirectly, when they referred to the refereeing process but not a specific referee comment that could be otherwise classified (eg, "a very thoughtful review", "authors responsive", "good revisions," "positive reviews").

When we examined the 603 written comments made by the editors following each manuscript discussion, we found that science, journalism, and writing were all important factors in the editors' opinions, regarding whether to proceed with acceptance or not [see Table 3]. Research design and methods (science) phrases were used most often, with importance to medicine (journalism), and comments related to the refereeing process (writing) next. The rankings of written comments were not meaningfully affected when we examined research articles only or accepted articles only.

**Table 3:** Coded Reasons to Proceed and Not to Proceed with Publication, as Noted by Editors in Phrases on Written Forms Completed for Each Manuscript

**Phrase code**	**Classification**	**Number of phrases written**
Research design & methods	Science	144 (23.9)
Important to medicine	Journalism	59 (9.8)
Peer review comments	Writing	51 (8.4)
Analysis	Science	49 (8.1)
Presentation & interpretation of results	Science	46 (7.6)
Interesting results or topic	Journalism	42 (7.0)
Power, sample size	Science	26 (4.3)
Population studied	Science	25 (4.1)
More data needed	Science	19 (3.2)
Measures used	Science	17 (3.0)
Writing	Writing	15 (2.5)
Novel/new	Journalism	14 (2.3)
Positive or negative results	Journalism	12 (2.2)
Quality of the data	Science	11 (2.0)
Other reasons	Science, journalism, writing	73 (12.1)

**Total written phrases**	**--**	**603 (100)**

## Discussion

Our goal was to identify new aspects of editorial decision-making that can be used to guide research on publication bias, particularly relating to the stage of the process where editors physically meet around a table and by teleconference to discuss the merits of the manuscripts presented to the group. We found that the discourse at manuscript meetings could be recorded and analyzed, using two means of word and phrase collection. One method was simple note-taking at the meeting, which, by its nature, is likely to be incomplete, for example because the note-taker did not hear or did not record all comments. The other method was a collection of a paper form on which editors were asked to list the reasons they were inclined to proceed to the next step in publication and reasons they were not. Neither of these methods uses standard qualitative research methodology, but instead combines qualitative and epidemiologic approaches. As far as we know, this methodology has not been employed before.

We developed our classification schema iteratively, with full knowledge of the phrases recorded. While our classification schema is only one of many possible perspectives, with overlapping categories, and the coding of individual phrases is subjective, our goal was to group editorial commentary in a way that would help future researchers to study the editorial process, rather than to identify "the truth".

By identifying the major topics of editorial discussion, our work contributes also to development of new variables for studying the publication process more generally. While a body of literature exists on factors involved in editorial decision making, the hypotheses tested in past studies (eg, whether publication is associated with statistical significance of results) have mainly reflected individual authors' opinions and experience regarding the decision-making process, and anecdotal rather than recorded evidence.

We did not collect any data on the manuscripts rejected before this stage, and cannot infer anything about the process leading to rejection. It is possible for example, that some reasons for rejection are not reflected in the discussion of manuscripts considered at the manuscript meetings.

Most of the discussion around the table at the 12 *JAMA *manuscript meetings concerned the science of research presented in the manuscripts. This corresponds indirectly to the findings of Olson and colleagues who found an association between acceptance for publication and factors positively associated with study quality, for reports of clinical trials submitted to *JAMA *[7].

Perhaps more interesting is the discussion related to what we categorized as "journalism" concerns. This included phrases conveying the concept that the topic or findings were "important," as well as related phrases such as "interesting" and those associated with the study findings. While there is nothing surprising about medical journal editors being attracted to important or interesting reports, nor about physician-editors attaching degrees of importance to studies likely to affect the care of patients, we now are able to see the context in which study results are discussed. We would expect, and indeed found, that comments in this category were less frequent than those related to science, given that if the science is shaky there would be little point in much further discussion.

A second aspect of the journalism category involved the editors' interest in maximizing strategic advantage for the journal and related readership issues. Phrases classified in the "strategic emphasis" category included "refer to Archives", "published elsewhere", "we want the whole thing, not just a salami slice", "similar paper in revision", "not for *JAMA*", "too long", "too specialized". Examination of the statement of *JAMA*'s aims reveals extensive language indicating that such journalistic features of articles are valued. It is understandable that biomedical editors choose manuscripts that match the stated mission of a journal, which includes an embedded code of values and beliefs. No matter what the intention, those beliefs are a "differential inclination," and may contribute to publication bias, broadly defined. In addition, publishing is a business with profit-making goals, which can indirectly influence a journal's scope and selection of manuscripts [11], for example selection of articles likely to be frequently cited and thus positively influence the impact factor.

Written summaries of reasons to proceed to the next stage toward acceptance or not, completed for each manuscript by the editors, reflected the combined importance of science, journalism, and writing considerations. Peer reviewer comments ranked high in reasons given by editors to proceed toward acceptance or not. Given that the manuscripts discussed at meetings are those still under consideration after two elimination rounds, and that referees are not necessarily experts and can sometimes be wrong, the continuing influence of peer reviewers is especially important. Our meeting notes also indicate a major role for peer review in editorial decision-making, with more than 25% (459/1773) of the spoken phrases referring to comments by the reviewers.

In the future, it would be useful to examine whether accepted and rejected manuscripts differ in the discussion content and written summaries. It would also be interesting to see whether similar proportions of peer reviewer comments mentioned in the discussions were positive and negative. Finally, it would be useful to compare the comments attributed to the peer reviewers with phrases used by the editors, to see if their comments reflect similar concerns.

This project provides insight into the process at one journal, focusing specifically on comments leading to requests for manuscript revision, acceptance, or rejection. This journal was selected because it has a tradition of willingness to participate in research about the peer review process [7]. Although we do not know whether the process at *JAMA *is generalizable to other journals, expansion of our thinking in this area will inform future research, which was our goal.

## Conclusion

We developed a classification of editorial discourse that provides insight into editorial decision-making and concepts that need exploration in future studies.

## Competing interests

DR is Deputy Editor (West) at JAMA

## Authors' contributions

KD conceived of, designed, and provided oversight of the project, and participated in the acquisition, analysis and interpretation of data. With CM, she drafted the manuscript and she coordinated subsequent edits and revisions. She serves as the project's guarantor. ES contributed to acquisition, analysis, and interpretation of the data, and to revision of the manuscript. CM contributed to the concept and design of the study, the acquisition, analysis and interpretation of the data. She also participated in drafting the manuscript and its finalization. DR contributed to the concept and design of the study, and to revision of the manuscript. All authors have read and approved the final manuscript.

## Pre-publication history

The pre-publication history for this paper can be accessed here:


